# In-depth Retrospective Review of Originally Negative Screening Mammograms from Women with Confirmed Breast Cancer

**DOI:** 10.5334/jbsr.2796

**Published:** 2022-11-11

**Authors:** Lieve Vandendaele, Svetlana Jidkova, Koen Van Herck, Tom Kimpe, Veerle Verschuere

**Affiliations:** 1 Universiteit Gent & CvKO, Centrum voor Kankeropsporing, BE; 2 Universiteit Gent & CvKO, Centrum voor Kankeropsporing, Data Science Institute UZ Gent, BE; 3 Universiteit Gent, BE; 4 Belgian Cancer Registry, BE; 5 Barco, BE

**Keywords:** breast cancer, screening, quality control, peer review, digital mammography

## Abstract

**Objectives:**

We aim to contribute to the assessment of the screening performance in Flanders (Belgium) and to identify valuable mammograms for subsequent studies and training.

**Materials and Methods:**

Initially negative prior screening mammograms (sMx) of 210 women with confirmed breast cancer detected by the Flemish screening programme between 2011–2013 were reviewed by a highly experienced radiologist. The review of the prior sMx was performed in three steps: 1) only prior mammograms available; 2) with index sMx (=subsequent positive sMx) present; 3) with index sMx and clinical information present.

**Results:**

The radiological review yielded 94 (45%) mammograms ‘without suspicious lesions’, 77 (37%) ‘with minimal signs in at least one breast’, and 39 (19%) ‘with clearly visible tumours’. In univariate analyses, the reclassification of prior sMx was significantly associated with the date of the prior sMx, the need for a third reader for arbitration, image quality and the detector system used (computed radiography versus direct readout digital radiography), and it was not associated with the interval between screening rounds, age at prior sMx, breast density, or tumour characteristics (<T2 versus ≥T2, in situ versus invasive). In multivariate analyses, the date of the prior sMx (p = 0.001), need for arbitration (p = 0.001) and image quality (p < 0.001) remained significantly associated with the reclassification.

**Conclusion:**

This retrospective review reclassified 19% of the sMx as clearly visible tumours. With this, the Flemish screening programme performs in accordance with similar studies. The sMx reviewed in this study, form a valuable set of mammograms for training and further research.

## Introduction

Breast cancer screening programmes have substantially increased the number of early detected cancers [1]. However, studies have made clear that current screening programmes only capture about 70% of all breast cancers that occur in participating women [2,3,4].

To improve cancer detection by mammography screening the European guidelines advise quality control using predefined performance indicators and quality assurance including review and training. An important performance indicator is rating interval cancers (breast cancers arising after a negative screening episode and before the next scheduled screening round). Performing a radiological review of prior screening mammograms (sMx) of interval cancers is part of the quality assurance and also an important teaching tool [1]. Screen-detected cancers have different characteristics than interval cancers [3,5], and it is therefore useful to also review the priors of screen-detected cancers in order to improve the programme’s quality [1,6].

This study comprises a review of confirmed breast cancer cases detected by the Flemish screening programme. The aims were to quantify the proportion of visible tumours on the prior sMx and to gather insight into associated variables that may hinder cancer detection, such as breast density, age, image quality, imaging technique, tumour size, type of tumour, need of arbitration, screening interval, and date of prior sMx. The study also aimed to identify a valuable set of sMx for training and subsequent studies.

## Materials and Methods

In the breast cancer screening programme in Flanders, biennial two-view mammographic screening is offered free of charge to women aged 50–69 years. Two radiologists (first and second reader) independently evaluate the screening mammograms, with third reader arbitration if needed.

Between 2009–2013, 254,350 women participated in the Flemish Breast Cancer Screening Programme. From this group, cases for review were selected based on the following inclusion criteria: 1) informed consent for use of data in scientific research, 2) participation in minimum two consecutive screening rounds, 3) a screening interval of 16–30 months, 4) the index sMx (latest sMx) in 2011, 2012, or 2013 resulted in a referral for further diagnostic workup confirming and correctly documenting breast cancer, 5) where the prior sMx (previous sMx) was considered negative, 6) where the index and prior sMx were digital and available in the PACS (Picture Archiving and Communication System) at the Centre for Prevention and Early Detection of Cancer. In total, 292 cases met these inclusion criteria. From those a predefined sample size of 210 was selected by standard SPSS algorithms for random selection.

The 210 prior sMx were thoroughly reviewed by a single, highly experienced radiologist (reading > 10,000 sMx/year since 2006). The review followed a stepwise procedure: 1) review of prior sMx, in the absence of other images or information, 2) review of prior sMx with index sMx (subsequent positive screening mammogram) present, and 3) review of prior sMx, where index sMx and clinical information on tumour localization and characteristics (size, type, and stage) from diagnostic follow up were present. All steps were performed per case in succession. The expert radiologist reviewed all prior sMx for the presence of malignancy, the image quality, and breast density. The reviewing radiologist was not informed of the purpose of the study.

Possible associations between relevant variables and the intermediate (step 2) or final classification (step 3) were studied in univariate (chi-square) and multivariate analyses (logistic regression). 1) Breast density (≤25%, 26–50%, >50%), 2) age (50–54, 55–59, 60–64, 65–69 years), 3) image quality (good/not good), and 4) imaging technique (CR: computed radiography or DR: direct readout digital radiography) were considered as relevant variables with a possible association with non-detection of a visible cancer. Also 5) tumour size (<T2 versus ≥T2), 6) type of tumour (in situ versus invasive), 7) the need of a third reader for arbitration during the original reading process of the prior sMx (arbitration, no arbitration), 8) the interval between prior and index screening (17–20, 21–24, 25–28 months), and 9) the date of screening of prior sMx (earliest, intermediate and latest tertile) were tested. Tertiles were used instead of screening years due to an imbalanced distribution of cases across calendar years (see Table 1).

**Table 1:** Descriptive analyses of sMx and tumour characteristics.


DESCRIPTIVE DATA	N	%

Total	210	100

**Age at prior sMx^1^**		

50–54 years	55	26.2

55–59 years	56	26.7

60–64 years	74	35.2

65–69 years	25	11.9

**Date of prior sMx**		

2009	29	13.8

2010	99	47.1

2011	82	39

**Interval between prior and index sMx**		

17–20 months	19	9

21–24 months	169	80.5

25–28 months	22	10.5

**Arbitration needed for prior sMx**		

No arbitration	189	90

Arbitration	21	10

**Digital technique of prior sMx**		

Computed Radiography (CR)	71	33.8

Direct readout digital Radiography (DR)	139	66.2

**Tumour size**		

<T2^2^	169	80.5

≥T2	37	17.6

Missing	4	1.9

**Type of tumour**		

In situ	29	13.8

Invasive	181	86.2

**Staging**		

Stage 0 (in situ)	26	12.4

Stage IA	95	45.2

Stage IB	13	6.2

Stage IIA	40	19

Stage IIB	12	5.7

Stage IIIA	7	3.3

Stage IIIC	5	2.4

Stage IV	8	3.8

Missing	4	1.9


^1^ sMx: screening mammogram. ^2^ T2: Tumour more than 2 cm but not more than 5 cm in greatest dimension.

Because of the limited number of clearly visible tumours in the intermediate and final classification, bootstrap validation with bias correction and accelerated bootstrap interval was performed. Statistical significance was set at p < 0.05.

In the multivariate analysis, the group of clearly visible tumours was first compared with the compound group of minimal and no signs, subsequently the group of clearly visible tumours was compared with the no signs group only.

## Results

### Descriptive characteristics of sMx

Table 1 lists data from prior and index sMx and diagnostic follow up.

The sMx dataset contained images of 102 left, 103 right, and 5 bilateral breast cancers.

### Expert review of prior sMx

The results of the expert review are summarized in Table 2.

**Table 2:** The results of the expert review of the prior sMx.


STEP 1 REVIEW OF PRIORS ONLY	N	%

Total	210	100

**Image quality of prior sMx^1^**		

Good	148	70

Not good technical physical	20	10

Not good positioning	28	13

Not good technical physical nor positioning	14	7

**Breast Density on prior sMx**		

0–25%	80	38.1

26–50%	62	29.5

51–75%	64	30.5

76–100%	4	1.9

**Step 1 Bi-RADS categories: Review of prior sMx**		

No lesion	98	46.7

Benign lesion(s)	41	19.5

Probably benign	47	22.4

Probably malignant	24	11.4

Malignant	0	0

Total	210	100

**Step 2 interim classification: Reviewing priors with index sMx available**		

Without suspicious lesions	97	46.2

Minimal signs	88	41.9

Clearly visible tumour	25	11.9

Total	210	100

**Step 3 final classification: Reviewing priors with index sMx and clinical information available**		

Without suspicious lesions	94	44.8

Minimal signs	77	36.7

Clearly visible tumour	39	18.6

Total	210	100


^1^ sMx: screening mammogram.

By reviewing prior sMx alone (step 1), 24 of the sMx (11.4%) were labelled ‘probably malignant’ and might have been referred. The intermediate classification (step 2), prior sMx with index sMx present, identified 25 cases (11.9%) with ‘clearly visible tumours’. The final classification of prior sMx (step 3), including the use of index images and clinical information, revealed 39 ‘clearly visible tumours’ (18.6%).

### Univariate analyses

The intermediate classification was significantly associated with the date of prior sMx (p =< 0.001) and the need of arbitration on the prior sMx (p = 0.002). The final classification was significantly associated with the date of the prior sMx (p =< 0.001); the need of arbitration (p = 0.004), also with the image quality (p = 0.004) and the detector system used (CR versus DR) (p = 0.036). See Table 3. More ‘clearly visible tumours’ were detected in older sMx, sMx that required arbitration, in sMx of inferior quality, and in those using CR-technique.

**Table 3:** Univariate analyses: Variables significantly associated with the interim or final classification after reviewing prior mammograms.


A. UNIVARIATE ANALYSES: VARIABLES SIGNIFICANTLY ASSOCIATED WITH THE INTERIM CLASSIFICATION (STEP 2) AFTER REVIEWING PRIORS WITH INDEX IMAGES PRESENT.					

VARIABLE & CLASSES	WITHOUT SUSPICIOUS LESIONS	MINIMAL SIGNS	CLEARLY VISIBLE TUMOURS	TOTAL	PEARSON CHI-SQUARE

	97	88	25	210	

**Need of arbitration on prior imaging**					0.002

No arbitration	93 (49%)	78 (41%)	18 (10%)	189	

Arbitration	4 (19%)	10 (48%)	7 (33%)	21	

**Date of prior imaging**					<0.001

Earliest tertile	32 (67%)	15 (31%)	1 (2%)	48	

Intermediate tertile	40 (51%)	31 (39%)	8 (10%)	79	

Latest tertile	25 (30%)	42 (51%)	16 (19%)	83	

**B. UNIVARIATE ANALYSES: VARIABLES SIGNIFICANTLY ASSOCIATED WITH THE FINAL CLASSIFICATION (STEP 3) AFTER REVIEWING PRIORS WITH INDEX IMAGES AND CLINICAL INFORMATION PRESENT.**					

**VARIABLE & CLASSES**	**WITHOUT SUSPICIOUS LESIONS**	**MINIMAL SIGNS**	**CLEARLY VISIBLE TUMOURS**	**TOTAL**	**PEARSON CHI-SQUARE**

	94	77	39	210	

**Need of arbitration on prior imaging**					0.004

No arbitration	90 (48%)	69 (37%)	30 (16%)	189	

Arbitration	4 (19%)	8 (38%)	9 (43%)	21	

**Date of prior imaging**					<0.001

Earliest tertile	31 (65%)	15 (31%)	2 (4%)	48	

Intermediate tertile	39 (49%)	27 (34%)	13 (17%)	79	

Latest tertile	24 (29%)	35 (42%)	24 (29%)	83	

**Image quality at the tumour side**					0.004

Good	76 (43%)	59 (38%)	21 (14%)	156	

Not good	18 (33%)	18 (33%)	18 (33%)	54	

**Detector system used**					0.036

Computed Radiography CR	29 (41%)	22 (31%)	20 (28%)	71	

Direct Readout Digital Radiography DR	65 (47%)	55 (40%)	19 (14%)	139	


### Multivariate analyses

When clearly visible tumours were compared to the compound group of minimal and no signs, the need of arbitration on the prior sMx (p = 0.005) and the date of the prior images (p = 0.044) were independently significantly associated with false negative clearly visible tumours in step 2 (i.e., only using prior and index images). When clearly visible tumours were compared only to the group of no signs, the significance level for the need of arbitration (p = 0.001) and date of priors (p = 0.004) appeared even higher.

In step 3, the final classification (i.e., with prior and index images and clinical information available), the need of arbitration (p = 0.001) and the date of the prior images (p = 0.006) were still independently significantly associated with false negative clearly visible tumours. Furthermore, the image quality was statistically significant (p < 0.001). These conclusions held, whether comparing to the compound group of minimal and no signs or only to the no signs group. See Table 4.

**Table 4:** Multivariate analyses: Variables associated with the interim or final classification after reviewing prior mammograms.


A. MULTIVARIATE ANALYSES: VARIABLES ASSOCIATED WITH THE INTERIM CLASSIFICATION (STEP 2) AFTER REVIEWING PRIORS WITH INDEX IMAGES PRESENT.						

VARIABLES & CLASSES	CLEARLY VISIBLE TUMOURS COMPARED TO NO OR MINIMAL SIGNS			CLEARLY VISIBLE TUMOURS COMPARED TO NO SIGNS		

	ODDS RATIO	95% CONFIDENCE INTERVAL	p-VALUE	ODDS RATIO	95% CONFIDENCE INTERVAL	p-VALUE

**Need of arbitration on prior images**			0.005			0.001

No arbitration	1			1		

Arbitration	4.85	(1.61–14.61)	0.005	16.65	(2.98–93.00)	0.001

**Date of prior imaging**			0.044			0.004

Earliest tertile	11.13	(1.39–88.93)	0.024	39.71	(3.43–459.09)	0.003

Intermediate tertile	5.75	(0.68–48.72)	0.109	12.30	(1.06–142.17)	0.045

Latest tertile	1			1		

**Image quality at the tumour side**			0.510			0.220

Good	1			1		

Not good	1.40	(0.52–3.78)	0.510	2.02	(0.66–6.20)	0.220

**B. MULTIVARIATE ANALYSES: VARIABLES ASSOCIATED WITH THE FINAL CLASSIFICATION (STEP 3) AFTER REVIEWING PRIORS WITH INDEX IMAGES AND CLINICAL INFORMATION PRESENT**.						

**VARIABLE & CLASSES**	**CLEARLY VISIBLE TUMOURS COMPARED TO NO OR MINIMAL SIGNS**			**CLEARLY VISIBLE TUMOURS COMPARED TO NO SIGNS**		

	**ODDS RATIO**	**95% CONFIDENCE INTERVAL**	**p-VALUE**	**ODDS RATIO**	**95% CONFIDENCE INTERVAL**	**p-VALUE**

**Need of arbitration on prior images**			0.001			0.001

No arbitration	1			1		

Arbitration	5.72	(1.99–16.43)	0.001	12.24	(2.80–53.52)	0.001

**Date of prior imaging**			0.006			0.001

Earliest tertile	11.30	(2.30–55.46)	0.003	29.13	(4.40–193.06)	<0.001

Intermediate tertile	5.66	(1.11–28.81)	0.037	10.13	(1.53–67.12)	0.016

Latest tertile	1			1		

**Image quality at the tumour side**			<0.001			<0.001

Good	1					

Not good	4.41	(1.96–9.34)				<0.001


All statistically significant associations were confirmed by bootstrap validation.

## Discussion

This review of a substantial set of ‘initially negative’ prior sMx resulted in 39 (19%) being labelled as ‘clearly visible tumours’. This result is in accordance with similar studies [6,7]. It concerns tumours missed twice during the normal screening procedure (by the first and second reader, or if arbitration was necessary, by the third reader and one of first two readers) and are therefore very valuable for training.

The 19% missed tumours cannot automatically be considered ‘screening errors’, for several reasons:

1. the proportion of cases with ‘clearly visible tumours’ based on image review alone was 1/3 lower, at 12%. The availability of clinical information is known to alter the reading outcome [8,9].
2. Even if we tried to reproduce the conditions of routinely assessing sMx in the screening programme, the radiologist’s attention was presumably triggered by the clustering of challenging image sets, the slightly different protocol form, the specific categorization, and the stepwise assessment for the review [3,9].
3. The normal response of the human mind to low probability events (i.e., the low prevalence of cancer in the sMx) can be a substantial contributor to false negative errors in breast cancer screening [4].

Therefore, the clustering of challenging sMx in this study may have affected the reader’s awareness and the results of the review.

The image quality was significantly associated with the final categorisation of clearly visible tumours. This confirms the importance of a good image quality and therefore requires special attention [1].

In order to obtain a sufficient number of prior sMx we had to include sMx from the early stages of digital mammography screening in Flanders. The ‘date of screening’ effect may reflect a learning curve for the radiologists involved in the screening programme.

In several studies, DR detector systems seem to be superior to CR detector systems, also in clinical screening performance. Often higher sensitivity is found with higher cancer detection rates and less interval cancers, especially in dense breasts [2,10,11].

Since this review was performed by a single – albeit highly experienced – radiologist, the results of this retrospective review could not be corrected for inter-observer variability. This is a major limitation of this study.

## Conclusion

The radiological review yielded 94 (45%) mammograms ‘without suspicious lesions’, 77 (37%) ‘with minimal signs in at least one breast’, and 39 (19%) ‘with clearly visible tumours’. These results are in line with similar studies.

The screening mammograms assessed in this review are valuable for training and subsequent studies.

## Data Accessibility Statement

All relevant documentation or data in order to verify the validity of the results presented is available, but not openly. Due to the nature of this research, participants of this study did not agree for their data to be shared publicly.

## Abbreviations

sMx: screening mammogram

CR: computed radiography

DR: direct readout digital radiography

PACS: Picture Archiving and Communication System

BI-RADS: Breast Imaging Reporting & Data System
